# P53 Status as a Predictive Biomarker for Patients Receiving Neoadjuvant Radiation-Based Treatment: A Meta-Analysis in Rectal Cancer

**DOI:** 10.1371/journal.pone.0045388

**Published:** 2012-09-25

**Authors:** Min-Bin Chen, Xiao-Yang Wu, Rong Yu, Chen Li, Li-Qiang Wang, Wei Shen, Pei-Hua Lu

**Affiliations:** 1 Department of Medical Oncology, Kunshan First People’s Hospital Affiliated to Jiangsu University, Kunshan, Jiangsu Province, People’s Republic of China; 2 Department of Surgical Oncology, Kunshan First People’s Hospital Affiliated to Jiangsu University, Kunshan, Jiangsu Province, People’s Republic of China; 3 Department of Oncology, Suzhou Municipal Hospital, Affiliated Suzhou Hospital of Nanjing Medical University, Suzhou, Jiangsu Province, People’s Republic of China; 4 Department of Gastroenterology,Xuzhou Chinese Medical Hospital Affiliated to Nanjing University of Chinese Medicine, Xuzhou, Jiangsu Province, People’s Republic of China; 5 Department of General Surgery, Wuxi People’s Hospital Affiliated to Nanjing Medical University, Wuxi City, Jiangsu Province, People’s Republic of China; Northwestern University Feinberg School of Medicine, United States of America

## Abstract

**Background:**

Numerous studies have yielded inconsistent results regarding the relationship between p53 status and the response to neoadjuvant radiation-based therapy in patients with rectal cancer. We conducted a meta-analysis to clarify the relationship between p53 status and response to radiation-based therapy in rectal cancer.

**Methods/Findings:**

A total of 30 previously published eligible studies including 1,830 cases were identified and included in this meta-analysis. Wild-type form of p53 status (low expression of p53 protein and/or wild-type p53 gene) was associated with pathologic response in rectal cancer patients who received neoadjuvant radiation-based therapy (good response: risk ratio [RR] = 1.30; 95% confidence intervals [CI] = 1.14–1.49; p<0.001; complete response RR = 1.65; 95% CI = 1.19–2.30; p = 0.003; poor response RR = 0.85; 95% CI = 0.75–0.96; p = 0.007). In further stratified analyses, this association remained for sub-groups of good and poor response in neoadjuvant radiotherapy (RT) setting, good and complete response in chemoradiotherapy (CRT) setting. And the association between response and the presence of p53 gene mutations was stronger than that between response and protein positivity.

**Conclusion:**

The results of the present meta-analysis indicate that P53 status is a predictive factor for response in rectal cancer patient undergoing neoadjuvant radiation-based therapy.

## Introduction

In 2011, it is estimated that 39,870 new cases of rectal cancer will occur in the United states. In the same year, an estimated 49,380 people will die from rectal and colon cancer comined [1]. Today the increasing use of neoadjuvant radiation-based therapy(it is mostly RT and CRT) and improvements in the quality of rectal cancer surgery, particularly the standardisation of total mesorectal excision (TME), the combination of this strategies is recommended as a standard procedure for treatment of locally advanced rectal cancer[2]–[4]. However, despite generally high response rates, a small proportion of patients fail to respond to neoadjuvant radiation-basedtherapy, or even progress during therapy. There is now substantial evidence that biological markers may be useful for identifying those patients who would benefit from neoadjuvant therapy [5].

To date, p53 is the most studied response predictor in rectal cancer [6]. It is a master gene in the stress response that plays an important role in cancer development. The p53 tumour suppressor gene is the most widely mutated gene in human tumorigenesis, with mutations occurring in at least 50% of human cancers [7]. p53 encodes a transcriptional activator whose targets may include genes that regulate genomic stability, the cellular response to DNA damage, and cell-cycle progression [8]. Preclinical studies have shown that wild-type p53 was required for radiation-induced cell death in mouse thymocytes [9]. Thymocytes carried p53-homozygous mutants could resistant to 5.0 Gy, and p53 heterozygous thymocytes were relatively resistant to the same dose of radiation; while wild-type p53 cells were highly sensitive to the same dose of radiation [9]. These results have been confirmed in models of colorectal cancer *in vitro* and *in vivo* [10], [11].

The use of p53 status as a biological marker to predict the response of rectal cancer to neoadjuvant therapy, however, is disappointing, and the findings to date have shown conflicting results [6], [12]–[15]. Several studies found that patients with wild-type form of p53 often had better responses to therapy than those with mutation p53 [16]–[19]. Other studies, however, evaluated p53 status in rectal cancer patients and drew different conclusions [12]–[15]. These conflicting results may be attributable to the limited detection power inherent in studies that test small subsets of patients. We therefore performed a meta-analysis of the value of p53 status for predicting response to neoadjuvant radiation-based therapy in rectal cancer.

**Table 1:** TRG classification and standard definition.

TRG classification	standard definition		
	poor response	good response	complete response
Residual tumor rate (%)	≥75%	<50%	0%
Dworak or Rodel [20], [21]	Grade 0–1	Grade 3–4	Grade 4
Mandard [23]	TRG 4–5	TRG 1–2	TRG 1
AJCC [24]	TRG 3	TRG 0–2	TRG 0
JSCCR [25]	TRG 0–1	TRG 2–3	TRG 3
Lowe [9]	1	3–4	4
Elsaleh [26], Kandioler [17] and Kelley [27]	NR/SD	CR+PR	CR
Scott [28]	SD	CR+PR1	CR
RCRG staging [29]	RCRG 2–3	RCRG 1	–

NR:no response; SD: stable disease; CR: complete response; PR: partial response.

## Materials and Methods

### Publication Search

PubMed, Embase, and Web of Science databases were searched (up to May 8, 2012) using the search terms: ‘TP53’, ‘p53’, ‘p53 protein’, ‘p53 mutation’, ‘17p13 gene’, and ‘rectal cancer’. All potentially eligible studies were retrieved and their bibliographies were carefully scanned to identify other eligible studies. Additional studies were identified by a hand search of the references cited in the original studies. When multiple studies of the same patient population were identified, we included the published report with the largest sample size. Only studies published in English were included in this meta-analysis.

**Figure 1:**
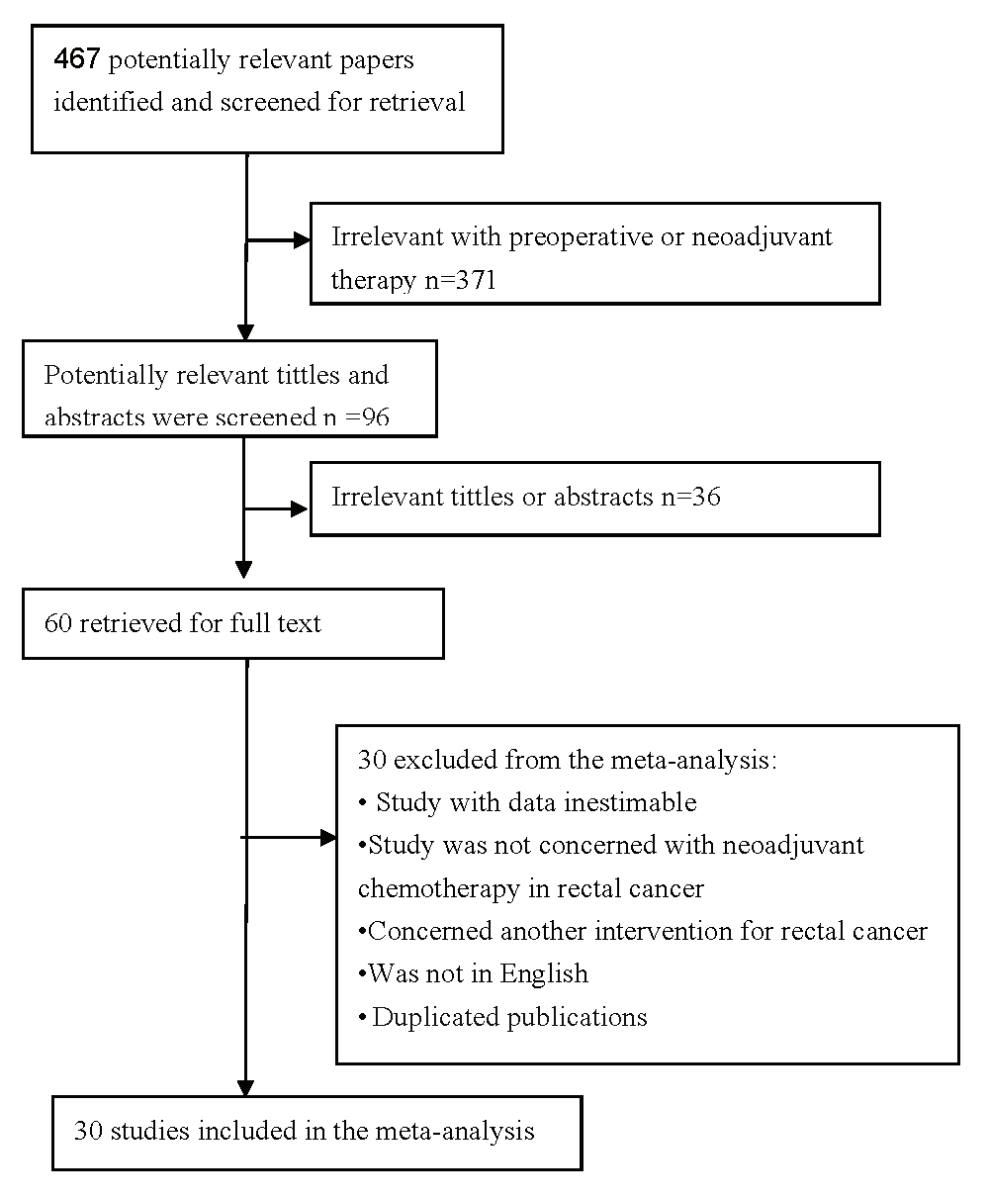
Improving the quality of reports of meta-analyses of randomized controlled trials; the Quality of Reporting of Meta-Analyses (QUOROM) statement flow diagram.

**Table 2:** Characteristics of studies included in the meta-analysis.

Author	Year	Country	N	Neoadjuvanttreatment	Detection	P53(%)*	Original TRGclassification	Provided information on pathologic response		
								poorresponse	goodresponse	completeresponse
Shinto [12]	2011	Janpan	96	CRT	protein	63%	Dworak	–	TRG 3–4	–
							JSCCR	TRG 0–1	–	–
Chen [13]	2011	USA	96	CRT	gene	54%	AJCC	TRG 3	TRG 0–1	TRG 0
Garcia [14]	2011	Spain	80	CRT	protein	61%	Rodel	grade 0/1	grade 3–4	grade 4
Chen [16]	2010	China	58	CRT	protein	40%	CR/PR/NR	NR	CR+PR	–
Brophy [15]	2009	Ireland	69	CRT	protein	50%	Mandard	TRG 4–5	TRG 1–2	TRG 1
Moral [30]	2009	Spain	39	CRT	protein	54%	–	No response	–	–
Jakob [31]	2008	Germany	22	CRT	protein	59%	Dworak	TRG 0–1	–	–
Zlobec [32]	2008	Switzerland	92	RT or CRT	protein	37%	CR/PR/NR	–	CR	CR
Negri [33]	2008	Italy	57	RT or CRT	protein	77%	CR/PR/NR	No response	CR+PR	–
Terzi [34]	2007	Turkey	37	CRT	protein	84%	Dworak	TRG 0–1	TRG 3–4	–
Kobayashi [35]	2007	Japan	52	CRT	protein	63%	Rodel	–	TRG 3–4	
Takeuchi [36]	2007	Japan	24	HRT	protein	63%	JSCCR	grade 0–1	grade 2–3	–
Sadahiro [37]	2007	Japan	96	RT or CRT	protein	61%	Mandard	TRG 4–5	–	–
Lopez-Crapez [38]	2005	France	70	RT or CRT	gene	50%	RCRG staging	RCRG 2–3	RCRG 1	–
Lopez-Crapez [38]	2005	France	70	RT or CRT	protein	56%	RCRG staging	RCRG 2–3	RCRG 1	–
Komuro [39]	2005	Japan	96	RT	protein	49%	JSCCR	grade 0–1	grade 2–3	–
Kelley [27]	2005	USA	50	CRT	protein	62%	CR/PR/NR	NR	CR+PR	CR
Suzuki [40]	2004	Japan	93	CRT	protein	54%	JSCCR	grade 0–1	grade 2–3	–
Suzuki [40]	2004	Japan	93	CRT	protein	54%	Mandard	TRG 4–5	–	–
Charara [41]	2004	USA	47	CRT	protein	45%	CR/PR/NR	–	CR	CR
Diez [42]	2003	Spain	73	RT	protein	73%	Lowe	1	3–4	4
Saw [43]	2003	Australia	58	RT or CRT	gene	43%	Mandard	–	TRG 1–2	–
Komuro [44]	2003	Japan	111	RT	protein	49%	JSCCR	grade 0–1	grade 2–3	–
Rebischung [45]	2002	France	86	RT	gene	51%	CR/PR/NR	No response		CR
Kandioler [17]	2002	Austria	64	RT	gene	45%	CR/PR/SD	SD	CR+PR	CR
Rodel [46]	2002	Germany	44	CRT	protein	45%	Rodel	–	grade 3–4	–
Nasierowska-Guttmejer [47]	2001	Poland	27	CRT	protein	54%	CR/PR1/PR2/SD	SD	CR+PR1	CR
Elsaleh [26]	2000	Australia	48	CRT	gene	35%	CR/PR/SD	–	CR+PR	–
Elsaleh [26]	2000	Australia	48	CRT	protein	40%	CR/PR/SD	–	CR+PR	–
Sakakura [48]	1998	Japan	28	HCRT	gene	50%	Rodel	grade 1	grade 3–4	–
Luna-Perez [18]	1998	Mexico	26	CRT	protein	54%	Residual tumor rate	≥75%	<50%	0%
Fu [19]	1998	Japan	49	RT	protein	49%	JSCCR	grade 0–1	grade 2–3	
Spitz [49]	1997	USA	42	RCT	protein	55%	Lowe	1	3–4	4

Hyperthermoradiation:HRT; hyperthermochemoradiotherapy:HCRT; N: number of patients analyzed;

* over expression of TP53 protein and/or TP53 gene mutation frequency(%); −:data not reported.

### Inclusion and Exclusion Criteria

Studies included in this meta-analysis had to meet all of the following criteria: (a) evaluation of p53 status for predicting the response to neoadjuvant radiation-based therapy in early-stage rectal cancer, locally-advanced rectal cancer, (b) described therapeutic pathological response, (c) retrospective or prospective cohort study, (d) inclusion of adequate data to allow the estimation of a risk ratio (RR) with 95% confidence intervals (95% CI), and (e) studies published in English. Reviews, letters to the editor, and articles published in books were excluded.

**Figure 2:**
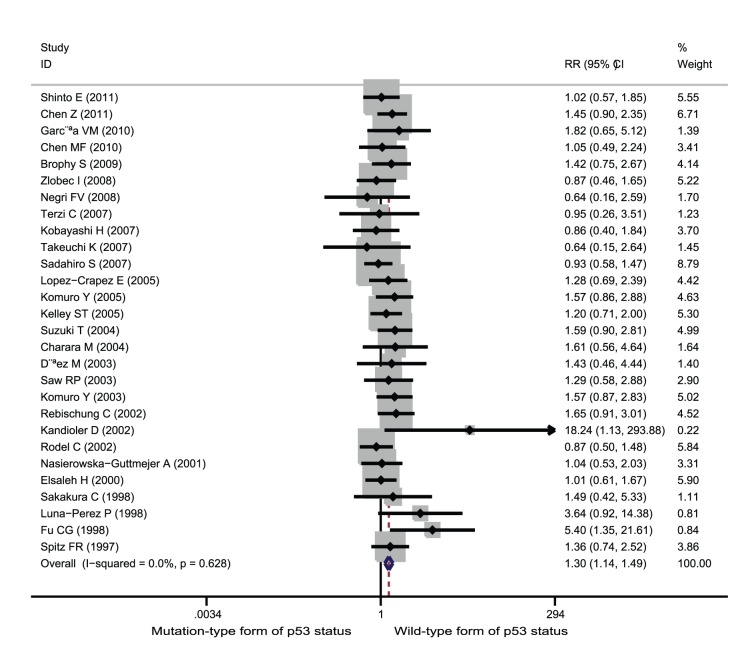
Forest plots of RR were assessed for association between p53 and good response among rectal cancer patients treated with neoadjuvant radiation-based therapy.

**Figure 3:**
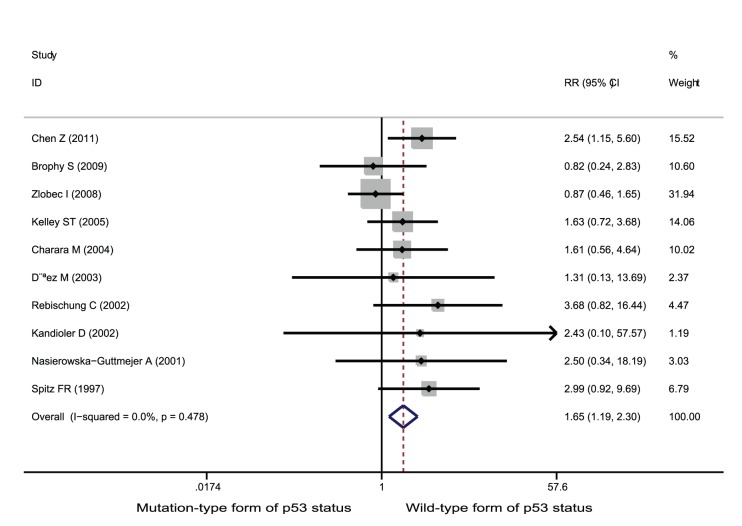
Forest plots of RR were assessed for association between p53 and complete response among rectal cancer patients treated with neoadjuvant radiation-based therapy.

**Figure 4:**
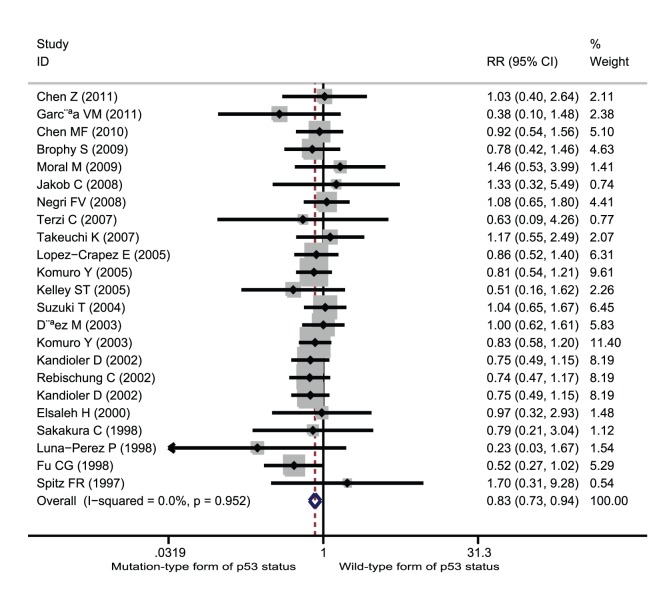
Forest plots of RR were assessed for association between p53 and poor response among rectal cancer patients treated with neoadjuvant radiation-based therapy.

### Data Extraction and Definitions

According to the inclusion criteria listed above, the following data were extracted for each study: the first author’s surname, publication year, country of origin, number of patients analyzed, treatment, types of measurement and over expression of TP53 protein and/or TP53 gene mutation frequency. Data on the main outcomes were entered in tables showing the pathological responses to radiation-based therapy with respect to p53 status. Information was carefully and independently extracted from all eligible publications by two of the authors (Chen and Wu). Any disagreement between the researchers was resolved by discussions until a consensus was reached. If they failed to reach a consensus, a third investigator (Lu) was consulted to resolve the dispute.

**Table 3:** Risk ratio for the association between wild-type form of TP53 and the response to neoadjuvant radiation-based radiotherapy.

comparison	complete response				good response				poor response			
	N	RR(95%CI)	p	Ph	N	RR(95%CI)	p	Ph	N	RR(95%CI)	p	Ph
all studies	10	1.65(1.19–2.30)	0.003	0.478	28	1.30(1.14–1.49)	0.000	0.628	24	0.85(0.75–0.96)	0.007	0.949
treatment												
RT	3	2.80(0.88–8.86)	0.081	0.764	7	1.90(1.44–2.51)	0.000	0.386	8	0.81(0.69–0.94)	0.007	0.783
CRT	6	1.92(1.26–2.91)	0.002	0.673	14	1.20(1.01–1.43)	0.043	0.860	12	0.87(0.68–1.12)	0.284	0.790
type of measurement												
protein	7	1.35(0.92–1.98)	0.124	0.562	23	1.18(1.02–1.36)	0.025	0.501	19	0.91(0.79–1.05)	0.191	0.729
gene	3	2.78(1.40–5.50)	0.003	0.909	7	1.48(1.15–1.91)	0.002	0.443	7	0.79(0.64–0.98)	0.033	0.994

Subgroup analysis was performed when at least two studies were in each subgroup.

N, number of studies; Ph, p value of Q-test for heterogeneity.

Two studies [26], [38] employed both protein and gene detection, we used the gene detection data, but also examined the protein detection data, and found similar results (data not shown).

One study [40] diagnosed complete response according to two criteria (JSCCR and Mandard AM), we used the data according to Mandard AM, but also examined the data according to JSCCR, and found similar results (data not shown).

**Figure 5:**
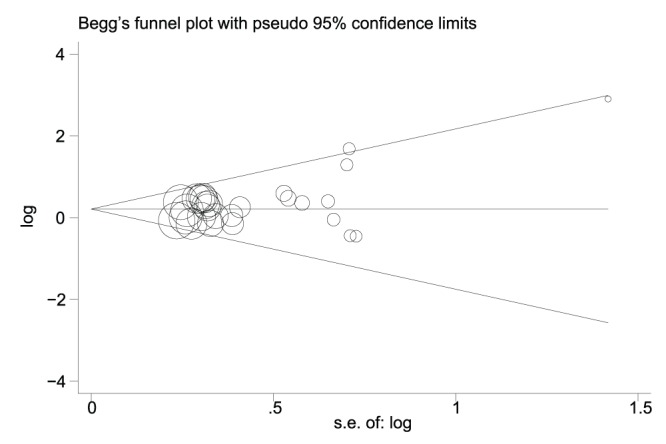
The funnel plot shows that there was no obvious indication of publication bias for the outcome of good response setting.

We used the definitions and standardizations for ‘p53’ and ‘response to radiation-based therapy’. For consistency, we used ‘p53 status’ to refer to both the gene and protein markers. Wild-type form of p53 status means patients with low expression of p53 protein and/or wild-type p53 gene. Pathologic response after neoadjuvant radiation-based therapy in different studies were according to different tumor regression grade (TRG) systems, most of the studies used TRG system described by Dworak et al. [20] and Rodel et al. [21], which categorize tumour regression in four grades, and TRG 2 and 3 determination is semiquantitatively defined as more/less than 50% tumour regression, respectively. Other grading systems have been proposed to categorize tumour regression in three grades where intermediate responders are grouped [22]. For consistency, we defined the pathologic response classification in the table 1. Briefly, poor response (residual tumor rate ≥75%); good response (residual tumor rate <50%); complete response (residual tumor rate = 0%).

### Statistical Analysis

RR with 95% CIs was used to estimate the association between p53 status and response to neoadjuvant radiation-based therapy in rectal cancer patients. Subgroup analyses were performed to evaluate the effects of different treatment regimens (CRT and RT) and different methods of p53 gene determination (protein and gene). The presence of statistical heterogeneity was assessed with Cochran’s Q test (considered significant for Ph<0.10). The pooled RR was calculated using a fixed-effects model (the Mantel–Haenszel method) or a random-effects model (the DerSimonian and Laird method), according to the heterogeneity. Funnel plots and the Egger’s test were employed to estimate the possible publication bias. We also performed sensitivity analysis by omitting each study or specific studies to find potential outliers. Statistical analyses were conducted using Stata (version SE/10; StataCorp, College Station, TX). p values for all comparisons were two-tailed and statistical significance was defined as p<0.05 for all tests, except those for heterogeneity.

## Results

### Eligible Studies

A total of 467 articles were retrieved by a literature search of the PubMed, Embase, and Web of Science databases, using different combinations of key terms. As indicated in the search flow diagram (Figure 1), 30 studies [12]–[15], [17]–[19], [26], [27], [30]–[49] reported at least one of the outcomes of interest and were finally included in the meta-analysis. The characteristics of the eligible studies are summarized in Table 2. Eighteen used neoadjuvant CRT and six used neoadjuvant RT, while five include both CRT and RT (Table 2), Twenty-five of the studies employed protein detection (including immunohistochemistry), seven employed gene detection (including genomic sequencing, Polymerase Chain Reaction-Single Strand Conformation Polymorphism[PCR-SSCP] etc.), two employed both methods (Table 2). The sample sizes in all the eligible studies ranged from 22–111 patients (median = 58 patients, mean = 61 patients, standard deviation [SD] = 4.73). Overall, the eligible studies included 1,830 patients. Eighteen of the studies were conducted in European or North American populations with mixed but mostly white participants (1,127 patients), whereas ten were conducted in East Asian populations (703 patients).

### Evidence Synthesis

Among the studies of rectal cancer patients who received neoadjuvant radiation-based therapy, 28 studies involving 1,769 patients contributed data on good response setting. Wild-type form of p53 status was significantly associated with improved good response among patients treated with neoadjuvant radiation-based therapy (RR = 1.30; 95% CI = 1.14–1.49; p<0.001, Figure 2). Ten studies involving 646 patients contributed data on complete response setting. Wild-type form of p53 status was significantly associated with improved complete response (RR = 1.65; 95% CI = 1.19–2.30; p = 0.003, Figure 3). Finally, 24 studies involving 1,478 patients provided information on poor response setting. Wild-type form of p53 status was significantly associated with decreases in poor response setting (RR = 0.85; 95% CI = 0.75–0.96; p = 0.007, Figure 4).

### Subgroup Analysis

Among the 30 studies in the neoadjuvant subgroup, 18 used neoadjuvant CRT and six used neoadjuvant RT, while five include both CRT and RT (Table 3). The results of the neoadjuvant CRT and RT were therefore calculated separately. Wild-type form of p53 status was associated with improved response in rectal cancer patients who received neoadjuvant CRT (good response: RR = 1.20, 95% CI = 1.01–1.43, p = 0.043, complete response: RR = 1.92, 95% CI = 1.26–2.91, p = 0.002), but not with poor response (RR = 0.91, 95% CI = 0.68–1.12, p = 0.284). Wild-type form of p53 status was associated with good response in neoadjuvant RT settings (RR = 1.90, 95% CI = 1.44–2.51, p<0.001), and with decreased poor response (RR = 0.81; 95% CI = 0.69–0.94; p = 0.007), but not with complete response(RR = 2.80, 95% CI = 0.88–8.86, p = 0.081).

Different measurements of p53 status (either by protein or gene detection) have been used to evaluate associations with favorable responses to neoadjuvant radiation-based therapy. We therefore calculated the associations using both protein and gene statuses of p53. The results of subgroup analysis are presented in Table 3. For gene detection, wild-type p53 gene was significantly associated with increased response (good response: RR = 1.48, 95% CI = 1.15–1.91, p = 0.002, complete response: RR = 2.78, 95% CI = 1.40–5.50, p = 0.003), and with decreased Poor response (RR = 0.79; 95% CI = 0.64–0.98; p = 0.033) among patients treated with neoadjuvant therapy. For protein-based detection, low expression of p53 protein was significantly associated with increased good response (RR = 1.18, 95% CI = 1.02–1.36, p = 0.025) among patients treated with neoadjuvant therapy, but not with complete response (RR = 1.35; 95% CI = 0.92–1.98; p = 0.124) and poor response (RR = 0.91; 95% CI = 0.79–1.05; p = 0.191).

### Publication Bias

Begg’s funnel plot and Egger’s test were used to estimate the publication bias of the included literature. The shapes of the funnel plots showed no evidence of obvious asymmetry (Figure 5), and Egger’s test indicated the absence of publication bias (p>0.05). Moreover, sensitivity analysis was carried out to assess the influence of individual studies on the summary effect. No individual study dominated this meta-analysis, and the removal of any single study had no significant effect on the overall results (data not shown).

## Discussion

The p53 status had been shown to play a pivotal role in the response to radiation-based therapy [6], [50]. Previous studies suggested that rectal cancers with p53 mutations might be either resistant or sensitive to neoadjuvant radiation-based therapy. However, the issue could not be resolved, because most of the available clinical reports involved small sample sizes, and the results were therefore unable to determine the value of p53 status for predicting the response to neoadjuvant radiation-based therapy. We therefore concluded that a meta-analysis was the best way of evaluating the association between p53 status and response to neoadjuvant radiation-based therapy in a large population.

The current meta-analysis of 30 studies systematically evaluated the association between p53 status and response to neoadjuvant radiation-based therapy in a large population. The results indicate that wild-type form of p53 status may predict good response rates to neoadjuvant therapy in patients with rectal cancer. Wild-type form of p53 status was associated with improved good and complete response, decreased poor response. Stratification according to different treatments showed that this association remained for sub-groups of good and poor response in RT, good and complete response in CRT, except for poor response in CRT and complete response in RT. Further stratification by gene detection revealed imprecise results, but amplification of the wild-type p53 gene was also associated with relevant increases in good and complete response, decreased poor response; however, although low expression of p53 was associated with relevant increases in good resonse, it was not associated with complete response and poor response. Gene detection was associated with advantages regarding response rates to neoadjuvant radiation-based therapy in patients with rectal cancer. The current meta-analysis suggests that p53 status as an independent predictive factor for neoadjuvant radiation-based therapy outcome in patients with rectal cancer, and gene detection may be a better assay to use in the evaluation of p53 status and sensitivity to neoadjuvant radiation-based therapy. Radiosensitive tumors could be identified by the detection of p53 status, a selective and individualized form of chemoradiation might be instituted. Novel molecular treatment strategies specifically designed to reactivate p53 within resistant tumors can be used as combined modality protocols to improve local response rate [5].

In interpreting our results of the current meta-analysis, some limitations need to be addressed. First, the meta-analysis may have been influenced by publication bias; although we tried to identify all relevant data and retrieve additional unpublished information, some missing data were unavoidable. Second, the studies used different measurements of p53 status (either protein or gene detection), and different tumor regression grade systems. Standardization is therefore of great importance for obtaining an accurate assessment of the clinical significance of p53 status. Although we made considerable efforts to standardize definitions, some variability in definitions of methods, measurements, and outcomes among studies was inevitable, which may lead to a misclassification bias. Third, Different measurements of p53 status (either byIHC or by DNA sequencing techniques) have been employed to evaluate association with favorable response to neoadjuvant radiation-based therapy. Cut-off values of p53 for both overexpression by IHC and gene amplification by PCR were not the same in each study, which might lead to inconsistent results between these studies. Therefore, standardization is particularly important when assessing p53 status (gene and protein), which will help to obtain accurate data with clinical significance. Fourth, our analysis was observational in nature, and we therefore cannot exclude confounding as a potential explanation of the observed results. Despite these limitations, this meta-analysis had several advantages. First, a large number of cases were pooled from different studies, and 1,830 subjects represent a sizeable number, significantly increasing the statistical power of the analysis. Secondly, no publication biases were detected, indicating that the pooled results may be unbiased.

This study is the first meta-analysis to assess the usefulness of p53 status for predicting the response of rectal cancer patients to neoadjuvant radiation-based therapy. Our data support p53 status as a useful predictive factor for assessing treatment response to neoadjuvant radiation-based therapy in rectal cancer patients. However, future studies with larger sample sizes, more advanced and accurate detection methods and more comprehensive study designs are required to confirm our finding.
